# Systematic review and meta-analysis of endoscopic ultrasound drainage for the management of fluid collections after pancreas surgery

**DOI:** 10.1007/s00464-022-09137-6

**Published:** 2022-03-04

**Authors:** Ali Ramouz, Saeed Shafiei, Sadeq Ali-Hasan-Al-Saegh, Elias Khajeh, Ricardo Rio-Tinto, Sanam Fakour, Andreas Brandl, Gil Goncalves, Christoph Berchtold, Markus W. Büchler, Arianeb Mehrabi

**Affiliations:** 1 Department of General, Visceral, and Transplantation Surgery, University Hospital Heidelberg, Im Neuenheimer Feld 420, 69120 Heidelberg, Germany; 2 Department of Gastroenterology, Digestive Oncology Unit, Champalimaud Foundation, Lisbon, Portugal; 3 Department of Digestive Surgery, Hepato-Pancreato-Biliary Surgery Unit, Champalimaud Foundation, Lisbon, Portugal

**Keywords:** Pancreatectomy, Collection, Ultrasonographic drainage, Drainage

## Abstract

**Background:**

The outcomes of endoscopic ultrasonography-guided drainage (EUSD) in treatment of pancreas fluid collection (PFC) after pancreas surgeries have not been evaluated systematically. The current systematic review and meta-analysis aim to evaluate the outcomes of EUSD in patients with PFC after pancreas surgery and compare it with percutaneous drainage (PCD).

**Methods:**

PubMed and Web of Science databases were searched for studies reporting outcomes EUSD in treatment of PFC after pancreas surgeries, from their inception until January 2022. Two meta-analyses were performed: (A) a systematic review and single-arm meta-analysis of EUSD (meta-analysis A) and (B) two-arm meta-analysis comparing the outcomes of EUSD and PCD (meta-analysis B). Pooled proportion of the outcomes in meta-analysis A as well as odds ratio (OR) and mean difference (MD) in meta-analysis B was calculated to determine the technical and clinical success rates, complications rate, hospital stay, and recurrence rate. ROBINS-I tool was used to assess the risk of bias.

**Results:**

The literature search retrieved 610 articles, 25 of which were eligible for inclusion. Included clinical studies comprised reports on 695 patients. Twenty-five studies (477 patients) were included in meta-analysis A and eight studies (356 patients) were included in meta-analysis B. In meta-analysis A, the technical and clinical success rates of EUSD were 94% and 87%, respectively, with post-procedural complications of 14% and recurrence rates of 9%. Meta-analysis B showed comparable technical and clinical success rates as well as complications rates between EUSD and PCD. EUSD showed significantly shorter duration of hospital stay compared to that of patients treated with PCD.

**Conclusion:**

EUSD seems to be associated with high technical and clinical success rates, with low rates of procedure-related complications. Although EUSD leads to shorter hospital stay compared to PCD, the certainty of evidence was low in this regard.

**Supplementary Information:**

The online version contains supplementary material available at 10.1007/s00464-022-09137-6.

Post-operative complications after pancreatic surgery can be life threatening and lead to major morbidity and mortality [1]. The most common complication after pancreatic surgery is peripancreatic fluid collection (PFC), which has been reported in up to 50% of cases [1–3]. PFC is caused by post-operative pancreatic leakage and occurs more often following central and distal pancreatectomy than following pancreaticoduodenectomy [2, 4–7]. Approximately 40% of PFCs need to be treated to avoid further complications [1, 2, 4]. Following pancreaticoduodenectomy, enzymes within the pancreatic fluid are active and can harm the adjacent vessels and organs, resulting in bleeding, tissue necrosis, and abscess formation. The main indications for PFC drainage are pain, infection, an increase in diameter of PFC, and obstruction of the biliary tract and gastric outlet [8].

PFCs often extend toward visceral organs and form irregular shapes, which make it difficult to drain the fluid [9, 10]. For decades, PFCs have been predominantly managed through conservative approaches, such as jejunal feeding, parenteral nutrition, sclerotherapy, and antibiotics [9, 10]. Surgical interventions have not been popular because of the increase in morbidity and mortality [4, 9–11]. Minimally invasive approaches, such as percutaneous drainage (PCD), showed better outcomes compared to surgical therapies and have promoted recovery [4, 9–11]. However, PCD has also been associated with a decreased quality of life due to the necessity of external drainage after discharge from hospital [12]. Furthermore, PCD increases the risk of persistent pancreas fistula [1, 13].

Recent studies have focused on endoscopic ultrasonography-guided drainage (EUSD) of PFC. EUSD has a high clinical efficacy and low morbidity and is considered the best option for managing pancreatic pseudocysts and PFCs [14–17]. Several advantages have been described for EUSD, such as the diagnostic possibility of fluid collection characterization. It also inflicts limited trauma on the surrounding tissue and shortens hospitalization [1, 13]. However, despite these advantages, the technical and clinical outcomes of EUSD have not been compared with those of other approaches, which prohibits the assessment of superiority. Larger sample sizes and longer follow-ups are needed to make reliable comparisons [1, 9, 13].

The current systematic review and meta-analysis evaluated the outcomes of EUSD in treatment of patients with PFC after pancreatic surgery and compared them with PCD.

## Materials and methods

This study was reported in accordance with the preferred reporting items for systematic reviews and meta-analyses (PRISMA) 2020 guidelines [18] and a PRISMA 2020 Checklist has been provided in Supplementary Tables 1 and 2. We performed two meta-analyses—A and B. In meta-analysis A, a systematic review and a single-arm meta-analysis of studies reporting the outcomes of EUSD in patients with PFC was performed. In meta-analysis B, the outcomes of PCD and EUSD in patients with PFC were compared.

### Eligibility criteria

The study question was formulated based on the population, intervention, comparison, outcome, and study design (PICOS) strategy. Studies were included in the study if they met the following criteria:

- *Population* Patients with post-pancreatectomy PFC
- *Intervention* EUSD to treat PFC after pancreatic resection
- *Comparator* None in meta-analysis A; PCD in meta-analysis B
- *Outcome* Technical success, time to drainage, clinical success, repeated drainage, hospital stay, and relevant perioperative data, including intra- and post-operative complications, as well as data regarding incidence of post-operative complications.
- *Study design* All study designs, except editorials and letter to editors.

To avoid analyzing the same patients more than once, the studies were thoroughly assessed and double publications and overlapping reports removed.

### Literature search

A systematic literature search in Medline (via PubMed) and ISI Web of Science was conducted using the following search terms: “(Endoscopic Surgical Procedures[tiab] OR Endoscopic drainage[tiab] OR Percutaneous transgastric drainage[tiab] OR transgastric drainage[tiab] OR Percutaneous transgastric irrigation drainage[tiab] OR Gastrointestinal Endoscopy[tiab] OR Minimally Invasive Surgical Procedures[tiab] OR Transmural drainage[tiab]) AND (Collection[tiab] OR Pancreas Collection[tiab] OR Pancreatic Collection[tiab] OR Fistula[tiab] OR Pancreas fistula[tiab] OR Pancreatic fistula[tiab]).” The search was not restricted to a specific study type or year of publication. The last query was performed in January 2022.

### Study selection and data extraction

After screening titles and abstracts in selected electronic databases, the full texts of appropriate studies were evaluated, and their data were extracted by three investigators (SS, SAHS, and AR) independently. Discrepancies among these investigators were resolved through discussions with a senior author (AM). For each study, the following data were extracted: study characteristics, patient characteristics, study quality, and the abovementioned outcome measures.

### Definition of extracted data

#### Demographic and pre-treatment data

Baseline data, including indication and type of primary pancreas surgery, PFC-related symptoms, and time to drainage, were obtained.

#### Post-treatment outcomes

Technical success was defined as successful insertion of the stents into the PFC endoscopically, as well as access to and drainage of the contents. Repeated drainage was defined as an unsuccessful initial drainage that needed additional interventions. Clinical success was defined as the resolution of the PFC and its associated symptoms. Procedure-related complications were stent migration, perforation, bleeding, sepsis, infection, and PFC recurrence. The duration of hospital stay after interventions was also recorded.

### Quality assessment

Two investigators (AR, SF) assessed bias in non-randomized studies using the ROBINS-I (Risk of Bias in Non-randomized Studies of Interventions) tool [19]. The risk of bias was assessed by considering the seven bias domains of the ROBINS-I tool, as follows: (1) confounding; (2) selection of participants; (3) classification of interventions; (4) deviations from intended interventions; (5) missing data; (6) measurement of outcomes; and (7) selection of the reported result. The overall risk of bias was determined as low if the study was judged to be at low risk of bias for all domains. Moderate risk of bias was considered, if the study was judged to be at some concerns in at least one domain. The risk of bias was considered serious if the study was judged to be at serious risk of bias in at least one domain or if the study was judged to have some concerns in multiple domains in a way that substantially lowered confidence in the result. In case of missing concordance, two senior authors (AM, RRT) resolved the issue. To summarize and visualize the risk-of-bias assessments outcomes, the *robvis* tool was used [20].

The certainty of evidence was assessed by applying “The Grading of Recommendations Assessment, Development and Evaluation (GRADE)” approach [21], and a summary of findings table was designed for all outcomes using the GRADEpro GDT software (GRADEpro GDT: GRADEpro Guideline Development Tool [software]. McMaster University, 2020 (developed by Evidence Prime, Inc.). Available from https://gradepro.org).

### Statistical analysis

For the single-arm meta-analysis, data proportions were analyzed using a random effects model. For the two-arm meta-analysis, dichotomous data were presented as odds ratios (OR) and continuous data as weighted mean differences (MD). Summary effect measures were presented along with their corresponding 95% confidence intervals (CIs). Statistical heterogeneity was assessed using *χ*^2^ and inconsistency (*I*^2^) analyses, and the threshold for heterogeneity was a *P* value lower than 0.05 or an *I*^2^ value greater than 50%. Publication bias was assessed using a funnel plot. The R software (version 4.0.1) and Meta package were used for data analysis.

## Results

**Fig. 1:**
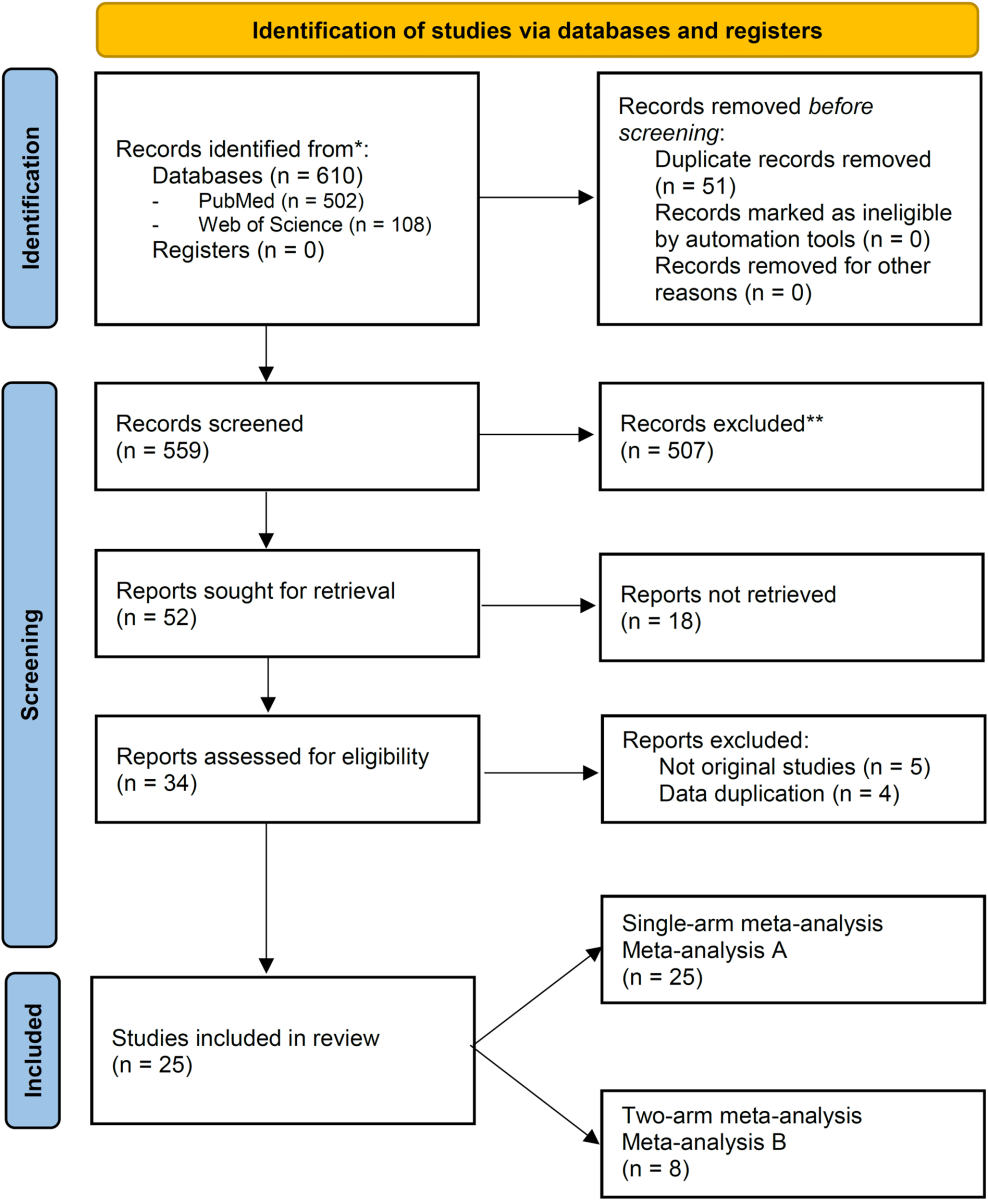
PRISMA flow-chart showing selection of articles for review

The literature search identified 264 articles, 25 of which met our eligibility criteria. These clinical studies reported 695 cases and were included in this meta-analysis [1, 10, 22–39] (Fig. 1). The articles were published between 2004 and 2021 and all had a retrospective non-randomized design. The baseline characteristics of the included studies are summarized in Table 1. Twenty-five studies (477 patients) were included in meta-analysis A and eight studies (356 patients) were included in meta-analysis B. Pre-procedural data of the patients and outcomes of the EUSD and PCD interventions are summarized in Tables 2 and 3, respectively.

**Table 1:** Baseline characteristics and pre-procedural data of the patients that underwent EUSD

Author, year	Country	Study design	Sample size		Gender (M/F)	Type of pancreas surgery				Meta-analysis	
			EUSD	PCD		Distal pancreatectomy	Pancreaticoduodenectomy	Central pancreatectomy	Other	A	B
Wang, 2021 [54]	China	Retrospective	15	–	5/10	8	7	–	–	x	–
Miranda, 2021 [36]	Germany	Case report	1	–	0/1	1	–	–	–	x	–
Storm, 2020 [26]	USA	Retrospective	75	–	–	63	3	–	9	x	–
Al Efishat, 2019 [24]	USA	Retrospective	39	39	39/39	48	20	6	4	x	x
Tamura, 2019 [25]	Japan	Retrospective	13	28	26/15	23	18	–	–	x	x
Caillol, 2019 [1]	France	Retrospective	35 (41)^†^	–	–	26	7	2	–	x	–
Donatelli, 2018 [23]	France	Retrospective	10	–	–	–				x	–
Ilie, 2018 [32]	Romania	Retrospective	2	–	2/0	–				x	–
Jürgensen, 2018 [55]	Germany	Retrospective	39	59	50/48	–				x	x
Futagawa, 2017 [22]	Japan	Retrospective	12	21	22/11	18	13	2	–	x	x
Mudireddy, 2017 [31]	USA	Retrospective	26	–	–	23	3	–	–	x	–
Chen, 2016 [56]	USA	Retrospective	40	–	22/18	–	40	–	–	x	–
Denzer, 2016 [9]	Germany	Retrospective	20	–	8/12	14	3	–	3	x	–
Tilara, 2014 [10]	USA	Retrospective	31	–	13/18	15	9	7	–	x	–
Kurihara, 2013 [35]	Japan	Retrospective	14	–	7/7	–	14	–	–	x	–
Kwon, 2013 [30]	USA	Retrospective	12	14	9/14	21	–	–	2	x	x
Azeem, 2012 [28]	USA	Retrospective	15	33	15/33	48	–	–		x	x
Onodera, 2012 [39]	Japan	Retrospective	6	18	17/7	6	18	–	–	x	x
Gupta, 2012 [27]	Belgium	Retrospective	23	–	–	–				x	–
Varadarajulu, 2011 [47]	USA	Retrospective	20	–	6/14	20	–	–	–	x	–
Ergun, 2011 [33]	Belgium	Retrospective	10	–	6/4	–	10		–	x	–
Grobmyer, 2009 [37]	USA	Retrospective	2	6	5/3	8	–	–	–	x	x
Varadarajulu, 2009 [29]	USA	Retrospective	10	–	6/4	10	–	–	–	x	–
Kahaleh, 2007 [34]	USA	Retrospective	5	–	–	–	5	–	–	x	–
Seewald, 2004 [38]	Germany	Retrospective	2	–	1/1	2	–	–	–	x	–

*EUSD* endoscopic ultrasonography-guided drainage, *PCD* percutaneous drainage

^†^Six patients with pancreatic enucleation were removed from further analysis, and totally 35 patients were included from this study

**Table 2:** Procedural data of the patients that underwent EUSD

Author, year	Country	Sample size		Symptoms						Time to drainage		
		EUSD	PCD	Fever	Nausea	Vomiting	Abdominal pain	Failure to intake diet	Enlarging collection	Acute (< 14 d)	Early (< 30 d)	Late (> 30 d)
Wang, 2021 [54]	China	15	–	x	–	–	x	–	–	–		
Miranda, 2021 [36]	Germany	1	–	–	–	–	–	–	–	1	–	–
Storm, 2020 [26]	USA	75	–	x	x	x	x	x	–	20	42	33
Al Efishat, 2019 [24]	USA	39	39	32	–	20	25	–	1	–		
Tamura, 2019 [25]	Japan	13	28	–						–		
Caillol, 2019 [1]	France	35^†^	–	23	–	–	35	–	2	–	–	–
Donatelli, 2018 [23]	France	10	–	–	x	x	–	–	x	–		
Ilie, 2018 [32]	Romania	2	–	–						–		
Jürgensen, 2018 [55]	Germany	39	59	–						–		
Futagawa, 2017 [22]	Japan	12	21	–						–	29	2
Mudireddy, 2017 [31]	USA	26	–	–						–		
Chen, 2016 [56]	USA	40	–	–						–		
Denzer, 2016 [9]	Germany	20	–	–	–	–	3	–	–	–		
Tilara, 2014 [10]	USA	31	–	13	1	1	26	–	1	–	17	14
Kurihara, 2013 [35]	Japan	14	–	–	–	–	2	–	–	–		
Kwon, 2013 [30]	USA	12	14	–						–		
Azeem, 2012 [28]	USA	15	33	–						–	36	12
Onodera, 2012 [39]	Japan	6	18	–						–		
Gupta, 2012 [27]	Belgium	23	–	–	–	–	x	–	–	–		
Varadarajulu, 2011 [47]	USA	20	–	14	–	–	20	–	–	–		
Ergun, 2011 [33]	Belgium	10	–	–						–		
Grobmyer, 2009 [37]	USA	2	6	–						–		
Varadarajulu, 2009 [29]	USA	10	–	6	–	–	10	–	–	–	2	6
Kahaleh, 2007 [34]	USA	5	–	–						–		
Seewald, 2004 [38]	Germany	2	–	–						–		

*EUSD* endoscopic ultrasonography-guided drainage, *PCD* percutaneous drainage

^†^Six patients with pancreatic enucleation were removed from further analysis, and totally 35 patients were included from this study

**Table 3:** Technical and clinical outcomes of the patients that underwent EUSD

Author, year	Country	Sample size		Technical success		Clinical success		Post-procedural complications (EUSD/PCD^a^)							Recurrence	
		EUSD	PCD	EUSD	PCD	EUSD	PCD	Total	Peri-procedural event	Hemorrhage	Stent migration	Abscess/ sepsis	POPF	Pain	EUSD	PCD
Wang, 2021 [54]	China	15	–	100%	–	93%	–	1	0	0	1	0	0	0	0	–
Miranda, 2021 [36]	Germany	1	–	100%	–	100%	–	0	0	0	0	0	0	0	0	–
Storm, 2020 [26]	USA	75	–	100%	–	93.3%	–	19	3	4	1	–	0	–	4	–
Al Efishat, 2019 [24]	USA	39	39	100%	97.4%	66.7%	59%	5/4	–	2/1	2/0	–	1/0	0/3	3	1
Tamura, 2019 [25]	Japan	13	28	100%	100%	100%	100%	1/3	0	0/1	0/2	–	–	–	1	0
Caillol, 2019 [1]	France	35^†^	–	100%	–	93%	–	9	–	3	4	5		–	0	–
Donatelli, 2018 [23]	France	10	–	100%	–	100%	–	–							0	–
Ilie, 2018 [32]	Romania	2	–	100%	–	100%	–	0	–	–	–	–	–	–	0	–
Jürgensen, 2018 [55]	Germany	39	59	–	–	85%	64%	0/4	–	0/x	–	0/x	–	–	–	–
Futagawa, 2017 [22]	Japan	12	21	92%	100%	92%	100%	0	–	–	–	–	–	–	4	–
Mudireddy, 2017 [31]	USA	26	–	100%	–	96%	–	–							0	–
Chen, 2016 [56]	USA	40	–	92.5%	–	87.5%	–	14	–	–	1	1	–	13	–	–
Denzer, 2016 [9]	Germany	20	–	100%	–	90%	–	0	–	–	–	–	–	–	1	–
Tilara, 2014 [10]	USA	31	–	100%	–	93%	–	2	0	1	0 (4^§^)	0	0	0	0	–
Kurihara, 2013 [35]	Japan	14	–	85.7%	–	85.7%	–	0	0	0	0	0	0	0	1	–
Kwon, 2013 [30]	USA	12	14	100%	100%	100%	79%	0/5	–	0/1	–	–	0/2	0/2	0	3
Azeem, 2012 [28]	USA	15	33	100%	97%	80%	81%	2/3	–	1/3	1/0	–	–	–	2	6
Onodera, 2012 [39]	Japan	6	18	100%	100%	100%	83%	0	0	0	0	0	0	0	0	0
Gupta, 2012 [27]	Belgium	23	–	100%	–	79%	–	–							3	–
Varadarajulu, 2011 [47]	USA	20	–	100%	–	100%	–	0	0	0	0	0	0	0	1	–
Ergun, 2011 [33]	Belgium	10	–	90%	–	80%	–	–							–	–
Grobmyer, 2009 [37]	USA	2	6	100%	100%	100%	67%	0	0	0	0	0	0	0	0	0
Varadarajulu, 2009 [29]	USA	10	–	100%	–	100%	–	1	0	0	1	0	0	0	0	–
Kahaleh, 2007 [34]	USA	5	–	100%	–	60%	–	–							–	–
Seewald, 2004 [38]	Germany	2	–	100%	–	50%	–	0	0	0	0	0	0	0	1	–

*EUSD* endoscopic ultrasonography-guided drainage, *PCD* percutaneous drainage, *POPF* post-operative pancreas fistula

^a^Number of complications divided between EUSD and PCD groups

^§^Number of stents

^†^Six patients with pancreatic enucleation were removed from further analysis, and totally 35 patients were included from this study

A detailed summary of the risk of bias for non-randomized two-arm studies using ROBINS-I tool is listed in Supplementary Fig. 1. The level assessment for each outcome was varied between moderate and serious risk of bias. Overall risk of bias for technical success, clinical success, post-procedural complications, and PFC recurrence were assessed as serious due to missing data and selection of results domain. Overall risk of bias for hospital stay was reported at moderate. In Table 4, a detailed level of evidence is summarized in GRADE assessment tables for non-randomized two-arm studies comparing EUSD and PCD in treatment of PFC after pancreas surgery, which provided low or very low level of certainty of evidence.

**Table 4:** GRADE assessment for EUSD vs. PCD in treatment of PFC after pancreas surgery—non-RCTs (comparative cohort studies)

Certainty assessment							Summary of findings				
Participants (studies) Follow-up	Risk of bias	Inconsistency	Indirectness	Imprecision	Publication bias	Overall certainty of evidence	Study event rates (%)		Relative effect (95% CI)	Anticipated absolute effects	
							With PCD	With EUSD		Risk with PCD	Risk difference with EUSD
Technical success											
258 (7 observational studies)	Serious^a^	Not serious	Not serious	Very serious^b^	None	⨁◯◯◯ Very low	157/159 (98.7%)	98/99 (99.0%)	OR 0.94 (0.14 to 6.13)	987 per 1000	1 fewer per 1000 (from 71 fewer to 11 more)
Clinical success											
356 (8 observational studies)	Serious^a^	Not serious	Not serious	Very serious^b^	None	⨁◯◯◯ Very low	167/218 (76.6%)	117/138 (84.8%)	OR 1.91 (0.96 to 3.82)	766 per 1000	96 more per 1000 (from 7 fewer to 160 more)
Post-procedural complication											
356 (8 observational studies)	Serious^a^	Not serious	Not serious	Very serious^b^	None	⨁◯◯◯ Very low	19/218 (8.7%)	8/138 (5.8%)	OR 0.69 (0.24 to 1.98)	87 per 1000	25 fewer per 1000 (from 65 fewer to 72 more)
Hospital stay											
117 (3 observational studies)	Serious^a^	Not serious	Not serious	Serious^c^	None	⨁⨁◯◯ Low	77	40	–		MD 3.84 lower (6.12 lower to 1.55 lower)
Recurrence											
225 (6 observational studies)	Serious^a^	Not serious	Not serious	Very serious^b^	None	⨁◯◯◯ Very low	10/138 (7.2%)	6/87 (6.9%)	OR 1.12 (0.27 to 4.76)	72 per 1000	8 more per 1000 (from 52 fewer to 199 more)

*CI* confidence interval, *MD* mean difference, *OR* odds ratio

*Explanations*: ^a^High risk of bias; ^b^Confidence interval overlaps no effect and is very wide; ^c^Confidence interval is very wide

### Technical success

**Fig. 2:**
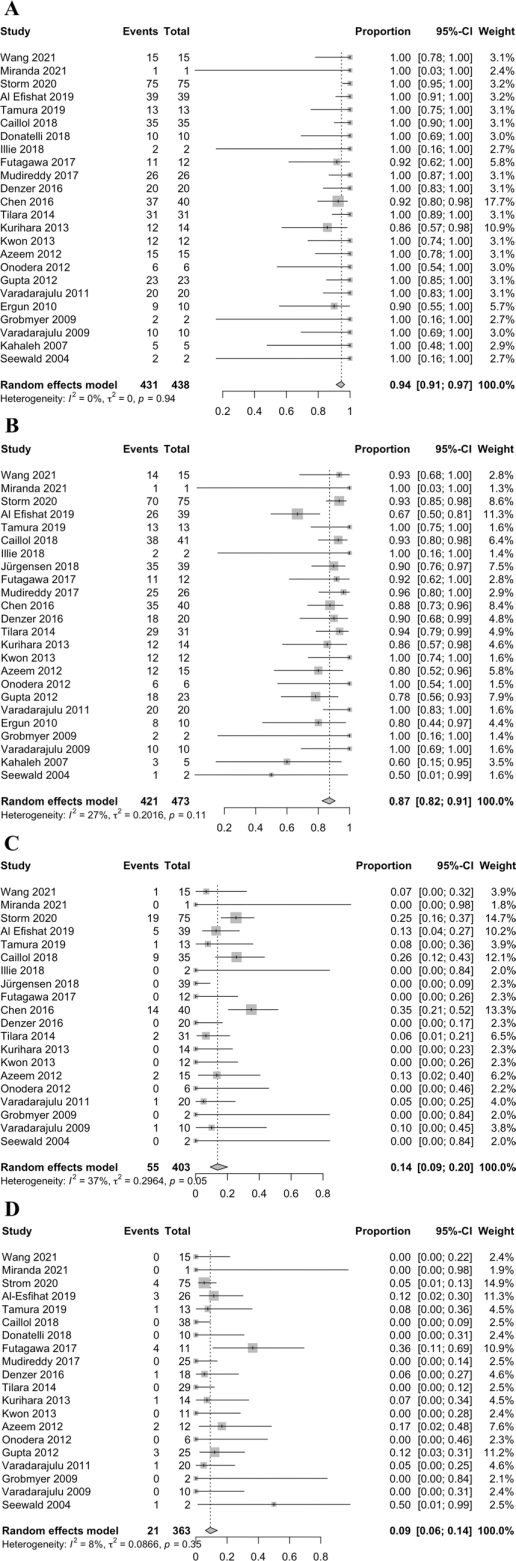
Forest plot of the single-arm meta-analysis (meta-analysis A) of EUSD outcomes; **A** technical success, **B** clinical success, **C** post-procedural complications rate, and **D** recurrence rate

**Fig. 3:**
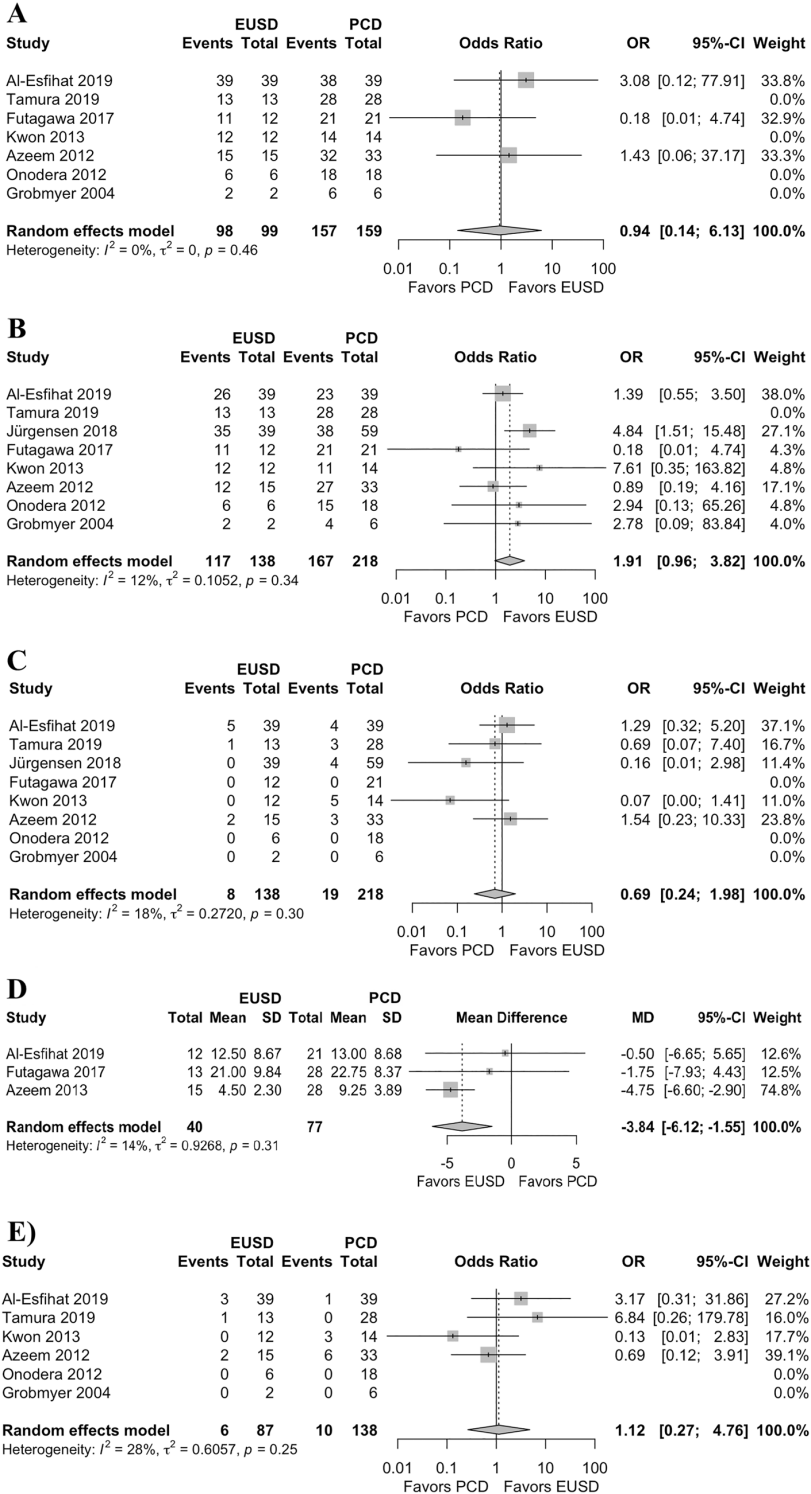
Forest plot of the two-arm comparison (meta-analysis B) of EUSD and PCD outcomes; **A** technical success, **B** clinical success, **C** post-procedural complication rate, **D** duration of hospital stay, and **E** PCF recurrence rate

In the single-arm meta-analysis of 24 studies, the technical success rate after EUSD was 94% (431/438 cases; 95% CI 91–97; *I*^2^ = 0%; Heterogeneity-P = 0.94) (Fig. 2A). The overall technical success rate reported in seven studies was 98.9% for EUSD and 98.7% for PCD, and the two-arm meta-analysis revealed no significant between two techniques in means of technical success (OR 0.94, 95% CI 0.14–6.12; *P* = 0.94; *I*^2^ = 0%; Heterogeneity-P = 0.45; Fig. 3A).

### Clinical success

The one-arm analysis of 24 studies indicated that the clinical success rate in the EUSD group was 87% (421/473 cases; 95% CI 82–91; *I*^2^ = 27%; Heterogeneity-P = 0.11) (Fig. 2B). In 16 studies, the authors reported the rate of re-intervention after EUSD due to unresolved symptoms or PFC. Of 246 patients, in 54 patients, the clinical circumstances after EUSD warranted repeated intervention, which revealed a rate of 23% (95% CI 17–31; *I*^2^ = 20%; Heterogeneity-P = 0.23).

In the comparative meta-analysis, eight studies reporting 356 cases gave the overall clinical success rate, which was 84.8% after EUSD and 76.6% after PCD. Albeit not significant, the meta-analysis showed higher rate of clinical success after EUSD compared to PCD (OR 1.9, 95% CI 0.95–3.81; *P* = 0.06; *I*^2^ = 12%; Heterogeneity-P = 0.34) (Fig. 3B).

### Post-procedural complications

Twenty studies describing 403 cases reported complications after EUSD. The one-arm analysis revealed a post-procedural complication rate of 14% (95% CI 9–20; *I*^2^ = 37%; Heterogeneity-P = 0.05) (Fig. 2C). Eight studies describing 356 cases compared the outcomes of EUSD (138 cases) and PCD (218 cases). The meta-analysis revealed a lower rate of post-procedural complications after EUSD, but this was not statistically significantly compared to rate of post-procedural complications after PCD (OR 0.69; 95% CI 0.24–1.98; *P* = 0.49; *I*^2^ = 18%; Heterogeneity-P = 0.30) (Fig. 3C).

### Hospital stay

In means of two-arm meta-analysis, the duration of hospital stay was reported in 40 patients with PFC after EUSD and in 77 patients with PFC after PCD. The meta-analysis showed that the length of hospital stay was significantly shorter after EUSD than after PCD (mean difference: − 3.84; 95% CI − 6.12 to − 1.55; *P* < 0.01, *I*^2^ = 14%; Heterogeneity-P = 0.31) (Fig. 3D).

### PFC recurrence

PFC recurrence after EUSD was reported in 21/363 patients from 20 studies. One-arm analysis showed that the incidence of PFC recurrence after EUSD was 9% (95% CI 6–14; *I*^2^ = 8%; Heterogeneity-P = 0.35) (Fig. 2D). In six studies reporting 193 cases, PFC recurrence was 6.9% after EUSD and 7.2% after PCD and these rates were not significantly different according to the two-arm meta-analysis (OR 1.12; 95% CI 0.27–4.76; *P* = 0.87; *I*^2^ = 28%; Heterogeneity-P = 0.25) (Fig. 3E).

## Discussion

In this study, we compared the outcomes of EUSD and PCD in patients with PFC after pancreatic surgery. Our single-arm meta-analysis showed that EUSD achieved a technical success rate of 94% and a clinical success rate of 87%. The complication rate was 14% with a PFC recurrence rate of 9% after EUSD. Our two-arm meta-analysis showed no significant difference neither in technical nor in clinical success rates between EUSD and PCD. However, patients had a significantly shorter hospital stay after EUSD compared to PCD. The rate of PFC recurrence was also similar between these two techniques. None of the included studies reported procedure-related mortality.

In a previous systematic review and meta-analysis, Mahon et al. reported a technical success rate of 97% and a clinical success rate of 93% after EUSD, which was comparable to the provided single-arm meta-analysis [13]. However, unlike our study, Mohan et al. observed a significantly higher clinical success rate after EUSD than after PCD. This discrepancy may be explained by the use of pooled data analysis to compare outcomes between groups in the Mohan et al. study as well as the inclusion of patients after all pancreatic procedures in their study [13]. We performed a two-arm meta-analysis with non-significant heterogeneity, which is a more reliable method of comparing EUSD with PCD. The rate of procedure-related complications after EUSD was 9.3% in the study by Mohan et al., which was similar to our findings. PFC recurrence after EUSD was 9.4% in the Mohan et al. study, which was comparable to our rate of 9%. This discrepancy may be explained by differences of the included studies and approaches to data analysis.

PFC that are suitable for endoscopic drainage are endoscopically accessible and located within 1 cm of the duodenal or gastric walls [40, 41]. Indications for EUSD include unusual location of the collection, small window of entry, non-bulging collections, coagulopathy, intervening varices, failed conventional transmural drainage, indeterminate adherence of PFC to the luminal wall, or suspected malignancy. EUSD is also a feasible option for draining PFCs with a septum, when PCD might be difficult. It has been suggested that PFC should be drained by EUSD at least four weeks after the surgery to allow a thick capsule to form around the collection [22]. Nonetheless, other studies have shown acceptable outcomes when EUSD was performed less than 2 weeks after surgery. In the current study, most patients (69%) received EUSD less than 4 weeks after surgery, which was confirmed by recent studies showing a trend to earlier application of EUSD after PFC diagnosis than previously reported. These findings suggest that PFCs can be drained by EUSD during the early post-operative period with excellent outcomes. EUSD has also proven to be feasible during the early post-operative period in patients with PFC after pancreaticoduodenectomy [39]. Despite the technical difficulty and anatomic variations, EUSD provides the opportunity to visualize anatomically important structures.

In addition to its technical feasibility and safety, EUSD has also been shown to improve quality of life and reduce the risk of infection [42]. Even in patients with infectious PFC, draining the fluid into the stomach via EUSD did not result in fever, gastroenteritis, or retrograde infection [22, 27]. Furthermore, EUSD prevents fluid and electrolyte loss, which can occur after PCD, and diminishes the risk of persistent collections and fistula [10, 12, 30, 43]. The current study was not able to show that EUSD had better outcomes compared to PCD in our study. Reported disadvantages of EUSD include inconvenient nasocystic drainage and repeated endoscopic interventions because of stent migration or incomplete drainage [44]. As a limitation of this technique, EUSD might not be feasible in all patients with PFC [45]. For example, the PFC needs to be in direct contact with the stomach for EUSD [14], so a pre-interventional computed tomography scan is advised to define the anatomic characteristics of the PFC.

EUSD outcomes are affected naturally by operator- and patient-dependent factors, such as the number and type of stents, physician’s experience, and extent of the PFC [46, 47]. Regarding stent type, double pigtail stents may prevent stent migration thereby reducing the risk of re-intervention [48, 49]. However, it may not be possible to insert two stents into some patients if the location or shape of the PFC is unsuitable [28], therefore appropriate patient selection is crucial [13]. In some cases, the PFC might contain solid debris from detached necrotic tissues. In these circumstances, EUSD might be useful not only for real-time detection of the debris through imaging but also for debriding the necrotic tissue and performing a direct necrosectomy, which is not possible via PCD [50, 51]. In patients with debris in the PFC, metal stents are thought to be superior to plastic stents due to their larger diameter [52, 53].

There are some limitations to the current study. The main weakness is the low number of two-arm studies and the small samples in the included studies. In addition, we were not able to carry out subgroup analyses for the type of pancreatic resections and for the type and number of stents. A further limitation is that the required data were not available in all studies, which reduced the power of the analysis. Besides, our analyses revealed shorter post-procedural hospital stay after EUSD compared to PCD; however, this can be significantly influenced by the primary surgical procedure and pre-procedural duration of stay, which might lead to bias in interpretation of outcomes of the analyses. Another weakness was the lack of randomized controlled trials that compared the outcomes of EUSD and PCD in patients with PFC.

In conclusion, the present study shows that EUSD is a safe and feasible approach to draining PFCs after pancreatic resections. EUSD provided a high technical and clinical success rate and a low rate of procedure-related complications and PFC recurrence. These encouraging features make EUSD an interesting option for treatment of PFC after pancreatic surgery. Satisfying outcomes can be achievable by meticulous patient selection and good technical experience. However, randomized trials with large patient cohorts are needed to comprehensively evaluate the advantages and disadvantages of this procedure compared with other drainage techniques.

## Supplementary Information

Below is the link to the electronic supplementary material.

Supplementary file1 (DOCX 767 kb)
